# Emergent Cesarean Section Due to Malaria: A Perioperative Challenge

**DOI:** 10.7759/cureus.88621

**Published:** 2025-07-23

**Authors:** Tanmai Bandarupalli, Reine Zbeidy

**Affiliations:** 1 Medicine, University of Central Florida College of Medicine, Orlando, USA; 2 Anesthesiology and Perioperative Medicine, University of Miami, Miami, USA

**Keywords:** falciparum malaria, general anesthesia in labor, malaria in pregnancy, nonendemic region, obstetric anesthesia

## Abstract

In a nonendemic setting, the confluence of malaria and pregnancy presents unique anesthetic challenges, particularly when the infection is undiagnosed at the time of an urgent cesarean section. This report involves a woman in her early 30s at 39 weeks of gestation with no prior health issues, who developed malarial symptoms upon returning from Haiti five months before. During labor, severe fetal heart rate decelerations necessitated immediate surgical intervention. Given the patient's febrile state, rapid induction with etomidate and succinylcholine was selected to minimize hemodynamic instability and secure the airway swiftly. Intraoperatively, close monitoring guided the systemic effects of malaria, such as hypotension and coagulation anomalies. Following delivery, empirical antimalarial treatment was initiated before the confirmatory diagnosis, considering the etiology endemic to the patient’s travel history. This case emphasizes the role of flexible, anticipatory anesthetic strategies in urgent obstetric procedures, specifically those involving complex infectious conditions.

## Introduction

The convergence of malarial infection and pregnancy poses a significant public health challenge, elevating risks for both the expectant mother and the fetus. Malaria is a worldwide, life-threatening infectious disease transmitted by the Plasmodium parasite. Pregnancy increases susceptibility to malaria and is associated with more severe symptoms and complications, including miscarriage, preterm delivery, and neonatal death. In some developing countries, maternal mortality rates reach as high as 1,100 per 100,000 live births, with malaria implicated in up to 23% of these cases [1]. In nonendemic settings such as the United States, diagnosis can be delayed due to the rarity of the disease, nonspecific symptoms, and limited clinician familiarity, particularly when recent travel history is unclear or overlooked. Additionally, anesthesia-specific considerations for malaria in pregnancy are underreported, in part due to the low incidence of perioperative encounters and the clinical focus on rapid obstetric management. This report presents a case of an urgent cesarean section in a patient with undiagnosed malaria at presentation, focusing on the perioperative anesthetic considerations and management strategies.

## Case presentation

Diagnostics

A G2P0010 woman in her early 30s at 39 weeks of gestation presented to a hospital in Florida with a one-week history of intermittent fever (peaking at 39.4°C), shaking chills, headache, and cough. Her fever exhibited a characteristic 48-hour cyclical pattern, with febrile spikes lasting four to six hours alternating with afebrile periods of 12-24 hours, marked by malaise but no rigors. This temporal pattern, combined with her travel to Haiti five months prior, raised clinical suspicion of malaria. Initial evaluation revealed tachycardia (HR = 111 bpm) but otherwise normal vital signs. Her recent travel history to Haiti was elicited during a review of systems for fever of unknown origin. She had an unremarkable medical history, and her pregnancy had been progressing without complications.

Additionally, there was no evidence of maternal anemia (Hb = 12.4 g/dL) or thrombocytopenia (platelets = 163,000/μL) (Table 1). Thrombocytopenia and coagulopathy are well-documented complications of *Plasmodium falciparum* infection and have been correlated with increased morbidity in both obstetric and nonobstetric populations. In this case, the patient’s preserved platelet count and normal coagulation profile suggested an absence of disseminated intravascular coagulation or consumptive coagulopathy, both of which are potential sequelae in severe malaria. On obstetric examination, the cervix was closed, there were no contractions, and fetal monitoring showed reactive tracing. The absence of uterine tenderness or ruptured membranes ruled out intra-amniotic infection as the source of fever. Given the suspicion of an infectious etiology, diagnostic tests, including blood and urine cultures, a chest X-ray, and a malaria blood smear, were ordered. Initial laboratory results revealed a white blood cell count of 11,700/μL, an activated partial thromboplastin time of 25.5 seconds, an international normalized ratio of 1.01, and a fibrinogen level of 400 mg/dL (Table 1).

**Table 1:** Laboratory results

Laboratory test	Patient result	Reference ranges
Hemoglobin	12.4 g/dL	Female: 12-16 g/dL
Platelet count	163,000/μL	150,000-400,000/μL
White blood cell count	11,700/μL	4,500-11,000/μL
Partial thromboplastin time/activated partial thromboplastin time	25.5 seconds	25-40 seconds
International normalized ratio	1.01	0.86-1.20
Fibrinogen	400 mg/dL	200-400 mg/dL

Treatment

At admission (day 0), the patient presented with fever and chills, exhibiting the previously described characteristics. Diagnostic blood cultures and malaria smears were obtained. Empiric broad-spectrum antibiotics, specifically vancomycin and piperacillin-tazobactam, were initiated for sepsis coverage, alongside general intravenous fluid resuscitation. Empiric antibiotics were initiated for febrile illness in pregnancy due to high sepsis risk, while concurrent malaria testing was pursued. Piperacillin-tazobactam was chosen based on institutional protocols for obstetric sepsis, covering polymicrobial intra-amniotic infections while avoiding aminoglycosides due to potential nephrotoxicity in volume-depleted patients.

Twelve hours after admission, the patient spontaneously entered labor. Approximately six hours into active labor (day 1), she developed severe, late fetal heart rate (HR) decelerations, prompting an emergent cesarean section while malaria testing was still pending. The decision to proceed was based on the prioritization of fetal rescue in the setting of suspected sepsis and non-reassuring fetal tracing. A rapid sequence induction and intubation were performed using etomidate and succinylcholine. Preoperatively, the patient was in compensated shock (blood pressure, BP = 95/68 mmHg; HR = 111 bpm), managed with 2 L of crystalloids and a phenylephrine infusion via a second large-bore peripheral IV at a starting rate of 50 mcg/minute, titrated to maintain a systolic BP above 90 mmHg and optimize uterine perfusion.

Intraoperative monitoring consisted of pulse oximetry, electrocardiography, and direct intra-arterial BP measurement. The fetus was delivered eight minutes after skin incision, and cord gas analysis showed mild metabolic acidosis with a cord pH of 7.25 and lactate of 3.1 mmol/L. The neonate's Apgar scores were 7, 9, and 9 at 1, 5, and 10 minutes, respectively. The patient remained hemodynamically stable throughout the procedure; her estimated blood loss was 800 mL, and her urine output was 100 mL. She was extubated in the operating room. Placental histopathology was not available, limiting the ability to confirm sequestration or directly correlate placental involvement with the fetal distress observed. In future cases, routine placental examination may provide additional diagnostic insight, as histology can reveal parasitized erythrocytes, fibrin deposition, or inflammatory changes consistent with placental malaria. The baby was transferred to the neonatal intensive care unit in stable condition, and neonatal blood smear negativity reflects the low maternal parasitemia (1%).

Following surgery and insertion of a central line, the patient was transferred to the intensive care unit. In the absence of confirmed microbiological results, clinical suspicion remained high, and quinidine with doxycycline was empirically initiated. Quinidine, though less commonly used today due to the wider availability of artemisinin derivatives, remains a recognized option for severe or suspected falciparum malaria in intravenous formulations. Doxycycline was included as an adjunct to enhance parasite clearance and reduce the likelihood of recurrence. Postoperatively (day 1), the blood smear results revealed *P. falciparum* infestation with a parasitic index of 1%. Although malaria smears typically return within four to six hours if prioritized as "stat," the result was not available until approximately 18 hours following sample collection due to institutional delays and lack of initial prioritization. Ultimately, these results prompted a transition in the treatment regimen from quinidine and doxycycline to chloroquine. She was initially administered 1 g, followed by 500 mg at 6, 24, and 48 hours. This regimen corresponds to 600 mg base initially and 300 mg base for each subsequent dose, as per the Centers for Disease Control and Prevention (CDC) guidelines for the treatment of uncomplicated *P. falciparum* malaria in chloroquine-sensitive cases.

Outcome and follow-up

The patient demonstrated a steady and complete recovery over the course of one week. After hospital discharge, the patient was monitored through a structured follow-up plan, including outpatient evaluations by infectious disease and obstetric specialists. At the two-week postpartum visit, the patient demonstrated full recovery from malaria, with resolution of all febrile episodes and no further systemic complications. Repeat blood smears confirmed complete parasitic clearance. She remained normotensive, afebrile, and reported no residual symptoms. The patient successfully resumed daily activities within four weeks of surgery. The incision site healed appropriately, with no signs of infection or dehiscence. Given her history of travel-acquired malaria, the patient was counseled on preventive strategies for future travels to endemic regions, including chemoprophylaxis recommendations per CDC guidelines. The neonate remained asymptomatic, with no evidence of congenital malaria, and continued to receive routine pediatric follow-up.

## Discussion

In this case, a pregnant woman residing in a nonendemic region presented with suspected malaria, highlighting the critical role of travel history in diagnosis. Despite the absence of hallmark malaria symptoms such as anemia or thrombocytopenia, her symptoms and history led to the identification of *P. falciparum* infection. Malaria during pregnancy, particularly due to *P. falciparum*, increases the risk of severe maternal and fetal complications.

This case exemplifies key diagnostic and management considerations for malarial infection in pregnancy, especially in nonendemic regions. Malaria is most commonly imported into the United States from endemic regions and can be caused by four Plasmodium species: *Plasmodium vivax*, *Plasmodium ovale*, *Plasmodium malariae*, and *P. falciparum* [2]. The clinical manifestations of malaria arise from its hematogenous spread, resulting in hemolysis. A hallmark symptom of this disease is hyperpyrexia, often presenting in a cyclical pattern every 48-72 hours. Furthermore, small vessel occlusion, which is a characteristic feature of malaria, can result in systemic hypoperfusion and hypotension. This patient’s symptoms, including febrile episodes, chills, headache, and hypotension, aligned with typical malaria presentations, though the diagnosis was less immediately apparent due to her residence in a nonendemic region. This further reinforces the value of a detailed travel history in the workup of febrile pregnant patients.

An additional diagnostic challenge was the absence of the common malaria-associated symptoms of maternal anemia or thrombocytopenia. Thrombocytopenia is particularly prevalent in adults experiencing severe falciparum malaria; however, it was absent in this patient, despite falciparum infestation in the blood smear [3]. The decision to initiate empirical treatment with broad-spectrum antibiotics, while awaiting definitive diagnosis, aligns with the best practices in managing undifferentiated febrile illness in pregnancy. Once Plasmodium species are identified, clinicians must remain vigilant of the associated complications, particularly in falciparum malaria (present in this case), which has the most serious sequelae, including acute lung injury, disseminated intravascular coagulation, renal failure, and coma [4]. Pregnancy increases susceptibility to serious infections and complications due to immunologic changes and the tendency of falciparum species to become sequestered in the placenta [5]. As a result, pregnant women face a significantly higher risk of death from severe malaria, ranging from two to ten times greater than that of nonpregnant individuals [5]. Additionally, malaria in pregnant women can double the likelihood of giving birth to a low-weight baby or experiencing stillbirth [6]. The possibility of the malaria parasite passing from the mother to the fetus is also a serious concern. In one study, 57% of pregnant women with malaria in their blood also had the parasite in their placenta, and 3.8% had it in the umbilical cord blood [7]. The key factor influencing the risk of transmitting malaria to the fetus is the severity of the infection at delivery, particularly when there is a high concentration of parasites in the peripheral blood and placenta [7]. If fetal transmission occurs, there is an increased risk of anemia, which was observed in 8%-33% of newborns in such cases [6]. In our case, the malaria blood smear of the baby was negative.

Regional anesthesia is generally favored in obstetrics over general anesthesia to circumvent the increased risks associated with difficult intubations and their associated complications. A 2020 study particularly highlights that in their group of emergency cesarean patients, among 403 patients receiving initial epidural anesthesia, only eight required conversion to general anesthesia [5]. In the context of this case, general anesthesia was selected due to the necessity of rapid action in response to the patient’s severe fetal distress and her acute febrile state from systemic sepsis, conditions perpetuated by malaria that warranted a swift airway management approach not typically pursued in standard obstetric procedures. Although there was no apparent evidence-based contraindication to epidural/spinal neuraxial anesthesia in cases of malaria, provided there was no thrombocytopenia or coagulopathy, general anesthesia was chosen due to the clinical urgency of the case and hemodynamic instability caused by fever, tachycardia, and hypotension. Additionally, general anesthesia also avoided the theoretical concern of neuraxial hematoma with thrombocytopenia, even though platelets were normal in this patient. The decision on anesthetic drugs for general anesthesia should focus on ensuring maternal hemodynamic stability and uterine perfusion. To minimize neonatal respiratory depression, rapid induction agents are preferable, choosing drugs like etomidate or ketamine for their beneficial cardiovascular effects [7].

## Conclusions

This case reinforces the importance of early recognition and prompt treatment, including empirical antibiotic therapy and specific antimalarial drugs guided by a high index of clinical suspicion. Such suspicion is rooted in maintaining a broad yet systematic differential when encountering febrile pregnant patients, especially in nonendemic areas. Clinicians should remain vigilant for signs such as cyclical fevers, travel history to endemic regions even months prior, thrombocytopenia, hemolysis, or unexplained fetal distress not accounted for by more common etiologies (e.g., chorioamnionitis). Further, this case highlights the need for improved turnaround time for malaria diagnostics, particularly when delays may influence obstetric decision-making. Although fetal rescue rightly took priority, earlier administration of antimalarial therapy may have reduced perioperative risks. Finally, the decision to proceed with general anesthesia in this scenario illustrates the challenges of managing acute infectious diseases in pregnancy, where both maternal and fetal outcomes hinge on rapid and multidisciplinary care.
